# Sensitivity of pretargeted immunoPET using ^68^Ga-peptide to detect colonic carcinoma liver metastases in a murine xenograft model: Comparison with ^18^FDG PET-CT

**DOI:** 10.18632/oncotarget.25514

**Published:** 2018-06-08

**Authors:** Fanny Foubert, Sébastien Gouard, Catherine Saï-Maurel, Michel Chérel, Alain Faivre-Chauvet, David M. Goldenberg, Jacques Barbet, Clément Bailly, Caroline Bodet-Milin, Thomas Carlier, Françoise Kraeber-Bodéré, Yann Touchefeu, Eric Frampas

**Affiliations:** ^1^ CRCINA, INSERM, CNRS, Université d'Angers, Université de Nantes, Nantes, France; ^2^ Hepato-Gastroenterology Department, Institut des Maladies de l’Appareil Digestif, University Hospital, Nantes, France; ^3^ Nuclear Medicine Department, ICO René Gauducheau Cancer Center, Saint Herblain, France; ^4^ Nuclear Medicine Department, University Hospital, Nantes, France; ^5^ IBC Pharmaceuticals Inc., Morris Plains, New Jersey, USA; ^6^ Immunomedics Inc., Morris Plains, New Jersey, USA; ^7^ GIP ARRONAX, Saint-Herblain, Nantes, France; ^8^ Radiology Department, University Hospital, Nantes, France

**Keywords:** carcinoembryonic antigen, colonic cancer, liver metastases, ^18^FDG-PET, immuno-PET

## Abstract

**Purpose:**

The aim of this study was to compare the performances pretargeted immunoPET ^68^Ga-PETimaging (^68^Ga-pPET) with anti carcino-embryonic antigen (CEA) and anti-histamine-succinyl-glycine (HSG) recombinant humanized bispecific monoclonal antibody (TF2) and ^68^Ga-labeled HSG peptide (IMP288) to conventional ^18^FDG-PET in an orthotopic murine model of liver metastases of human colonic cancer.

**Methods:**

Hepatic tumor burden following intra-portal injection of luciferase-transfected LS174T cells in nude mice was confirmed using bioluminescence. One group of animals was injected intravenously with TF2 and with ^68^Ga-IMP288 24 hours later (n=8). Another group received ^18^FDG (n=8), and a third had both imaging modalities (n=7). PET acquisitions started 1 hour after injection of the radioconjugate. Biodistributions in tumors and normal tissues were assessed one hour after imaging.

**Results:**

Tumor/organ ratios were significantly higher with ^68^Ga-pPET compared to ^18^FDG-PET (*P*<0.05) with both imaging and biodistribution data. ^68^Ga-pPET sensitivity for tumor detection was 67% vs. 31% with ^18^FDG PET (*P*=0.049). For tumors less than 200 mg, the sensitivity was 44% with ^68^Ga-pPET vs. 0% for ^18^FDG PET (*P*=0.031). A strong correlation was demonstrated between tumor uptakes measured on PET images and biodistribution analyses (r^2^=0.85).

**Conclusion:**

^68^Ga-pPET was more sensitive than ^18^FDG-PET for the detection of human colonic liver metastases in an orthotopic murine xenograft model. Improved tumor/organ ratios support the use of pretargeting method for imaging and therapy of CEA-expressing tumors.

## INTRODUCTION

Colorectal cancer (CRC) is the third most frequently diagnosed cancer with about 1 million new cases and more than 500,000 deaths worldwide annually. Metastases develop in at least 50% of patients, most commonly in the liver [1]. In case of metastatic disease, the 5-year survival is less than 15% but may reach 60% when all lesions are resected [2]. CRC staging is based on anatomical imaging, such as computed tomography (CT) and magnetic resonance imaging (MRI). However, the sensitivity of these techniques for the detection of infra-centimetric tumors is less than 60% [3]. Fluorine-18 fluorodeoxyglucose (^18^FDG) positron emission tomography (PET) is highly sensitive for the detection of liver metastases and the diagnosis of CRC recurrence [4]. For the planned resection of metastatic lesions, FDG-PET can provide additional information to characterize and/or identify new potential lesions [5]. However, ^18^FDG-PET is hampered by a limited sensitivity for tumors less than 10 mm and lacks specificity in case of inflammatory or infectious lesions [6, 7].

To detect lesions, immuno-targeting strategies using intravenously injected radiolabeled antibodies as well as their fragments have been proposed many years ago. Optimal tumor imaging requires high tumor uptake and low retention of activity in normal tissues. Due to the slow blood clearance of IgG antibodies, the selective uptake in tumor lesions can be masked by excessive blood-pool or normal tissue activities. New targeting strategies have thus been investigated for the past 20 years, favored by advances in molecular engineering [8].

The pretargeted strategy has been developed to further improve the combination of high-binding, selective antibodies with higher tumor-to-blood ratios. In such a strategy, a nonradioactive bifunctional antibody with specificity for both a tumor antigen and a small molecule is first injected to selectively localize the tumor. Once sufficient blood clearance of the unbound fraction of the bifunctional antibody is achieved, the radiolabeled small molecule is injected and captured by the pretargeted antibody at the tumor, resulting in higher binding specificity and higher tumor-to-blood ratios compared to directly-labeled antibody targeting [9].

Carcinoembryonic antigen (CEA or CD66e) is a cell-surface glycoprotein over-expressed in a number of tumors, including more than 90% of colorectal cancers (CRC) [10]. Therefore, it became a favored target antigen for radioimmunolocalization of CRC. A novel pretargeting system uses a bispecific trivalent antibody against CEA and the histamine-succinyl-glycine (HSG) hapten called TF2, recently developed by the dock-and-lock (DNL) technology. The activity carrier consists of a DOTA-di-HSG peptide, IMP288 [11, 12]. This DOTA compound may be labeled with a variety of radioactive isotopes, such as ^111^In, ^99^Tc or ^68^Ga for imaging, but also ^90^Y or ^177^Lu for therapy in the so-called pretargeted radioimmunotherapy strategy [13–15]. ^68^Ga is a positron emitter suitable for PET imaging. Its 68-minute half-life is appropriate with the pharmacokinetics of the radiolabeled peptide and short enough to limit radiation exposure. Our study aimed at comparing standard ^18^FDG-PET imaging to pretargeted immuno-^68^Ga-PET (^68^Ga-pPET) imaging with TF2 and ^68^Ga-IMP288 in a preclinical orthotopic murine xenograft model of human colonic liver metastases.

## RESULTS

### PET imaging

**Figure 1:**
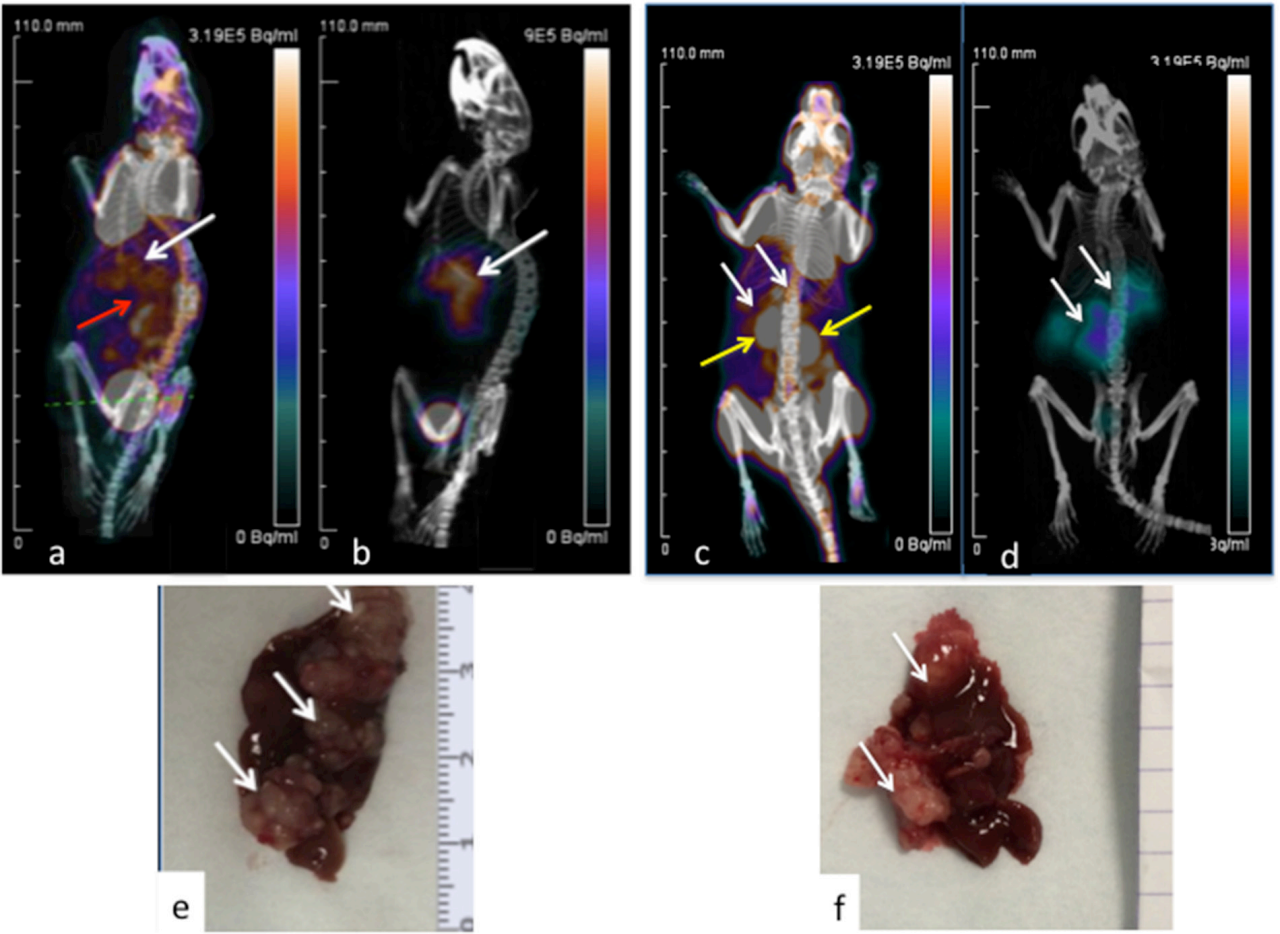
PET imaging of hepatic LS174T metastases (white arrows) in two mice 3D Volume Rendering. ^18^FDG PET-CT **(a, c)**. Images obtained 1h after intravenous injection of 13 MBq of ^18^FDG. Corresponding ^68^Ga-pPET-CT **(b, d)**. Images obtained 1 hour after IV injection of 10.1 MBq of pretargeted ^68^Ga-IMP288. Kidneys (yellow arrows), hepatic and bowel uptakes (red arrow). Corresponding macroscopic liver photography after dissection **(e, f)**. Images c and d correspond to the mouse illustrated in Figure 1 with bioluminescence.

On the ^18^FDG-PET images, the tumor uptake was hidden by the background uptake. Main normal uptakes were measured in heart, muscles and kidneys. Tumor discrimination was much better in ^68^Ga-pPET images, as adequate contrast was obtained between the high tumor uptake and the low normal tissue uptake (Figure 1).

**Figure 2:**
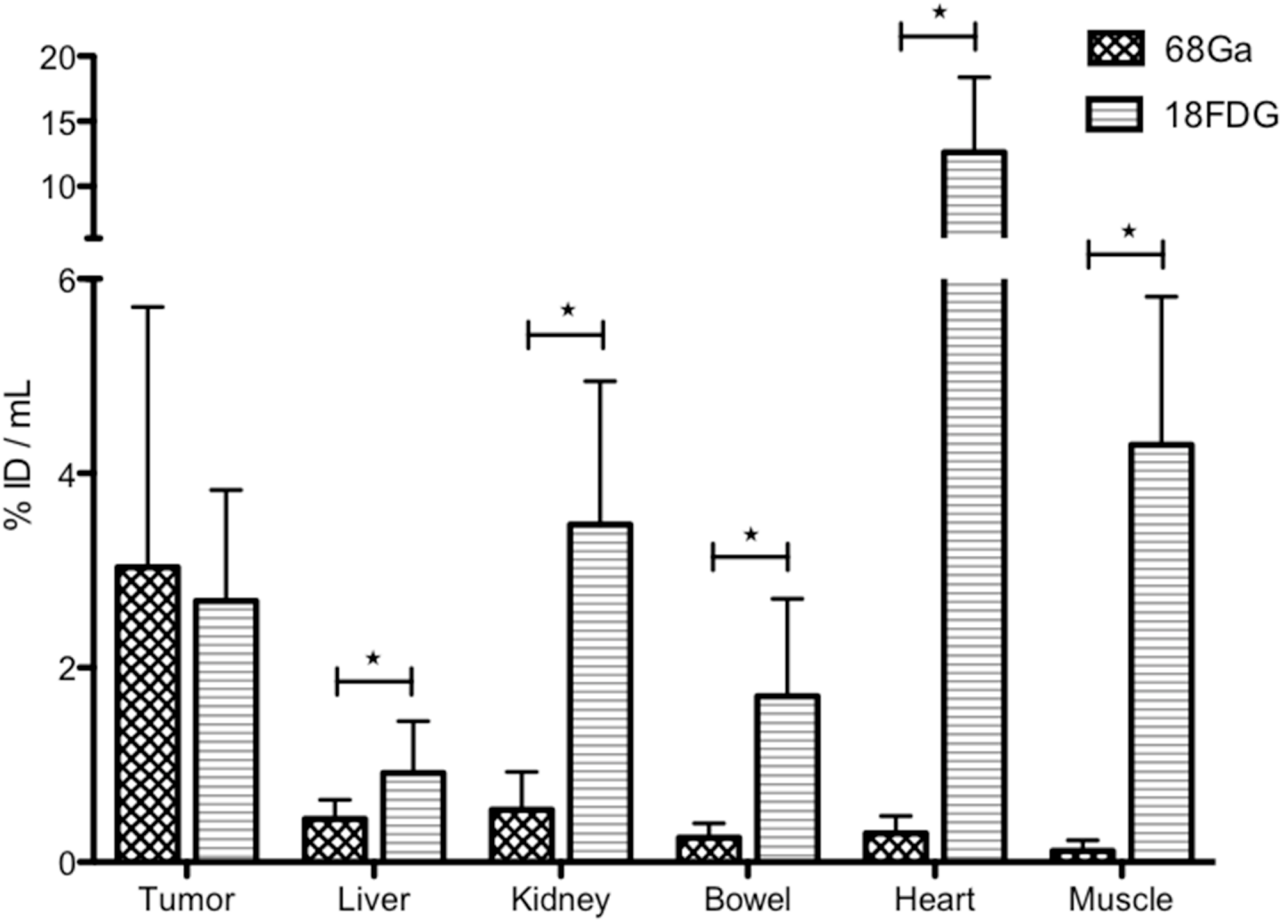
PET images analysis Comparison of uptakes obtained in ^68^Ga-pPET and ^18^FDG PET. For normal organs, values are obtained from a ROI drafted around the organ (for well- limited organs such as heart and kidney) or on the organ (for poorly-limited organs, such as liver, bowel or muscle) in each of the 2 selected images (most intense uptake). For tumors, the highest uptake in the tumor is obtained from a constant ROI of 2 mm^3^ drawn around the hottest spot.

Uptake measured in PET (in Bq/mL) from ROI on each tumor and each normal organ was analyzed, taking into account the injected activity and the physical decay of radionuclide. Normal organ uptake (in %ID/mL) was statistically higher in ^18^FDG-PET (n=16) versus ^68^Ga-pPET (n=15) (*P*<0.005). For tumor uptake, the difference between ^18^FDG-PET and ^68^Ga-pPET was not statistically significant (*P*=0.649) (Figure 2). Normal liver uptake in negative control mice («control without graft» imaged by ^68^Ga-pPET and «control without TF2» imaged in ^68^Ga-pPET) were 0.48 and 0.34 %ID/mL respectively, comparable to those of grafted mice imaged with ^68^Ga-pPET.

**Figure 3:**
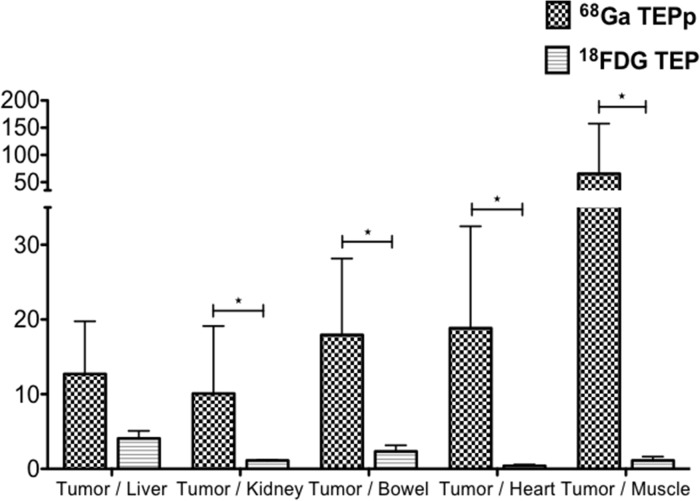
PET images analysis Intrahepatic tumor to non-tumor ratios with ^68^Ga-pPET and ^18^FDG PET.

To compare imaging contrast, tumor/organ ratios were calculated for both imaging modalities (Figure 3). Only mice with unequivocal tumor imaging were included in this analysis. Tumor/organ ratios in ^68^Ga-pPET (n=10) were statistically higher than tumor/organ ratios in ^18^FDG-PET (n=5) (*P*=0.001). Tumor/liver ratios were higher with ^68^Ga-pPET compared to ^18^FDG-PET (12.7 +/- 7.07 versus 4.08 +/- 1.01), although the difference was not statistically significant (*P*=0.099).

### Imaging performance

**Figure 4:**
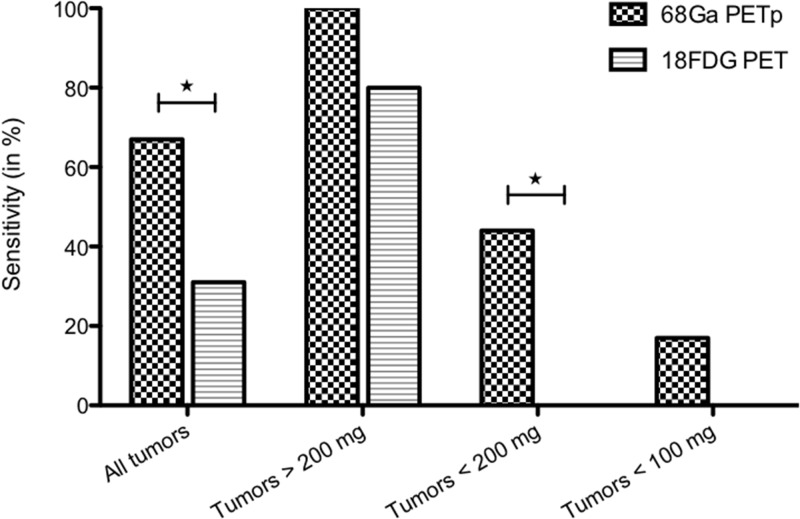
^68^Ga-pPET and ^18^FDG PET sensitivities, according to tumor weights

^68^Ga-pPET sensitivity for tumor detection was 67% versus 31% for ^18^FDG-PET (*P*=0.049). For smaller tumors less than 200 mg, the sensitivity was 44% with ^68^Ga-pPET versus 0% with ^18^FDG-PET (*P*=0.031) (Figure 4). The tumor detection threshold in ^68^Ga-pPET was 100 mg, while it was more than 600 mg in ^18^FDG PET.

### Biodistribution

**Figure 5:**
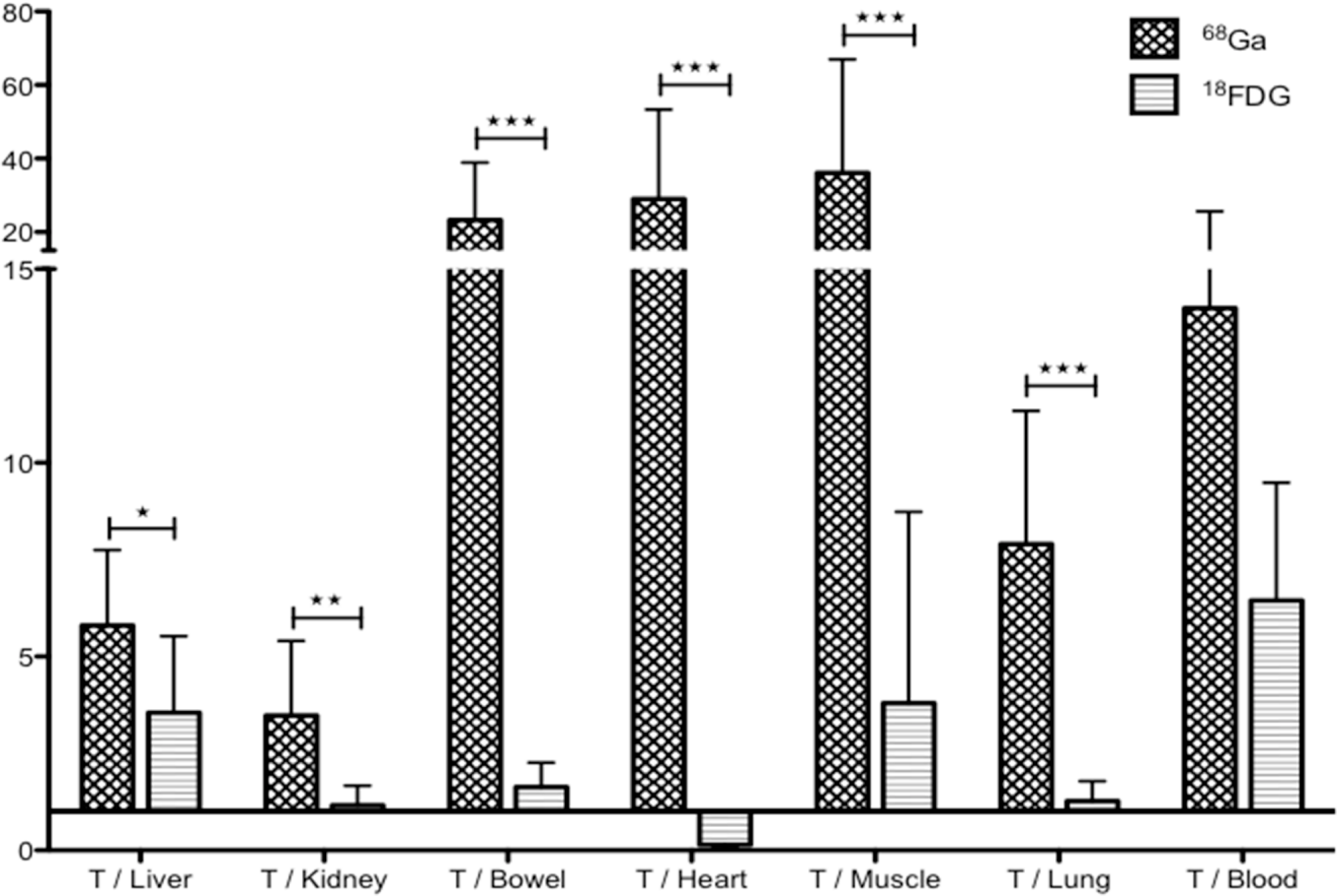
Biodistribution analyses Comparison of tumor/organ ratio in ^68^Ga-pPET and ^18^FDG-PET. (^*^: *P*=0.012, ^**^: *P*=0.0021, ^***^: *P*<0.0005).

In ^18^FDG-PET (n=13), 2 mice were not analyzed due to a breach of protocol. For 3 mice, tumors were not counted because no macroscopic tumor was detected for dissection. In ^68^Ga-pPET (n=8), the organ « bowel » was excluded from analysis for one mouse, because the measured activity was aberrant, probably due to a contamination of the counting tube. Uptakes were significantly higher in all organs with ^18^FDG-PET (P<0.05) except for tumors (Table 1). Tumor/organ ratios were higher in ^68^Ga-pPET compared to ^18^FDG-PET, with significant differences except for the tumor/blood ratios (*P*=0.515) (Figure 5). Data were comparable for healthy organs in the other model of subcutaneous tumors of medullary thyoid carcinoma.

**Table 1:** Biodistribution analyses

Tissue	^68^Ga pPET	^18^FDG-PET
	(n= 8)	(n=13)
Tumor	5.50 ± 0.96 (n=8)	7.61 ± 1.53 (n=10)
Blood ^*^	0.63 ± 0.19	1.21 ± 0,20
Liver ^**^	0.95 ± 0.15	2.26 ± 0.33
Kidney ^***^	1.88 ± 0.37	6.57 ± 0.77
Intestine ^***^	0.30 ± 0.10	4.99 ± 0.52
Lung ^***^	0.75 ± 0.14	5.77 ± 0.50
Muscle ^***^	0.35 ± 0.14	5.93 ± 1.78
Spleen ^***^	0.47 ± 0.07	4.52 ± 0.37
Skin ^***^	0.72 ± 0.25	3.08 ± 0.17
Brain ^***^	0.15 ± 0.06	5.74 ± 0.49
Heart ^***^	0.28 ± 0.08	61.57 ± 10.53
Bone ^***^	0.32 ± 0.09	3.85 ± 0.40
Stomach ^***^	0.43 ± 0.12	3.43 ± 0.45

(^*^: p=0.043, ^**^: p= 0.0018, ^***^: p= 0.0004).

Comparison of tumor and organ uptake in ^68^Ga-pPET and ^18^FDG-PET. (^*^: *P*=0.043, ^**^: *P*=0.0018, ^***^: *P*<0.0004).

**Figure 6:**
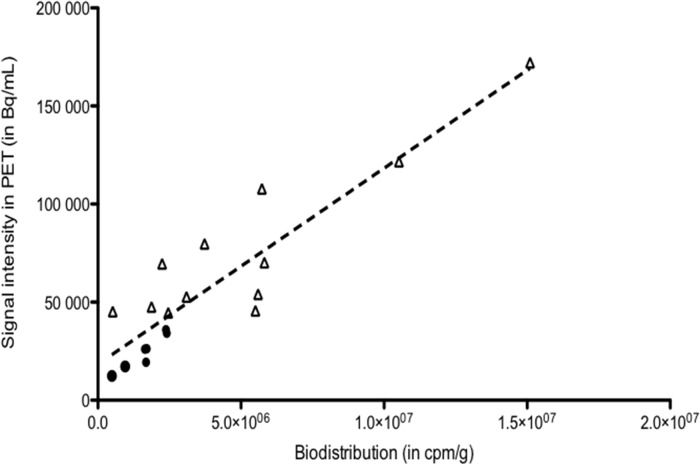
Correlation between biodistribution data and PET uptake for tumors (n=18). r^2^ = 0.85, *P*<0.0001

Figure 6 shows the correlation observed between biodistribution and PET uptake results (r^2^ = 0.85 and *P* < 0.0001).

## DISCUSSION

Diagnostic of intra- and extra-hepatic metastases for therapeutic management remains crucial and challenging. Current diagnostic imaging of CRC liver metastases mainly relies on CT, MRI and ^18^FDG-PET. ^18^FDG-PET is a highly sensitive noninvasive imaging modality for the detection of such lesions. Nevertheless, detection by ^18^FDG-PET is directly related to size, with reported sensitivities falling from 88% to 50% for lesions < 10 mm [7], and is still hampered by non-specific uptake, such as in inflammation.

Our study demonstrated that the novel pretargeted immuno-PETmodality was more sensitive than ^18^FDG-PET for human colonic liver metastases detection in an orthotopic murine model. PET image contrast was higher with pretargeted ^68^Ga-peptides PET than with ^18^FDG-PET, allowing the detection of smaller tumors.

Pretargeting is one way to improve tumor uptake and tumor to normal organ uptake ratios by reducing the molecular size of the radioactive agent and using a non-radiolabeled bispecific antibody, such as TF2. TF2 is a humanized bispecific monoclonal antibody produced by the Dock-and-Lock® technology, containing two anti-CEA Fab fragments and another Fab fragment binding the HSG hapten component of the IMP288 hapten peptide [11]. Compared to previously used bispecific antibodies with a single binding site for the tumor antigen and another for the hapten, this bivalency is expected to increase tumor accretion [16]. TF2 selectively and rapidly accumulates in the tumor, within 2 to 6 hours and also clears rapidly from blood and body in less than 24 hours. IMP288 is a DOTA-conjugated tetrapeptide containing two HSG hapten structures, also clearing rapidly from blood within an hour.

Uptakes in tumor reflecting tumor accretion were not statically different between ^68^Ga-pPET and ^18^FDG-PET. However ^68^Ga-pPET images analyses showed high tumor/organ ratios, due to low normal tissue uptake. By contrast, in ^18^FDG-PET imaging, background activity remains high despite measures used to optimize images, such as general anesthesia, fasting and warming of mice [17, 18]. The fast clearance of unbound TF2 allow rapid binding of labeled peptide to TF2 and rapid renal clearance of excess radiolabeled peptide [19–22]. These properties explain the low background activities observed in ^68^Ga-pPET.

Despite below 1 %ID/mL on biodistribution data, liver background remained significant in ^68^Ga-pPET. However, the tumor/liver ratios remained 3 times higher in ^68^Ga-pPET than ^18^FDG. Hypotheses for this liver background include a combination of intrahepatic blood uptake in such a highly vascularized organ, free ^68^Ga elimination, even if the free fraction of injected ^68^Ga is less than 5 % in our study and the ^68^Ga-IMP288 complex was shown to be stable for more than 4 hours in a previous study [23] and additional non-specific binding of TF2 to liver parenchyma.

In this study, we demonstrated that ^68^Ga-pPET was more sensitive than ^18^FDG-PET, especially for the detection of tumors smaller than 200 mg. For larger tumors, ^18^FDG-PET performance is well known, tumor cell metabolism being sufficient to provide a detectable uptake in PET. Thus, for tumor less than 200 mg, ^18^FDG-PET performance is surpassed by ^68^Ga-pPET. However, the sensitivity of ^68^Ga-pPET remained low for tumors less than 100 mg (17% versus 0% with ^18^FDG-PET). Those small tumors most frequently measured less than 2 mm in diameter. The PET intrinsic spatial resolution of 1.6 mm and the ^68^Ga positron range of 2.4 mm are probably the physical limits of ^68^Ga-PET detection in this situation.

Intrahepatic tumor grafting by means of an inoculation via the portal vein is an effective and well-controlled method to produce liver metastases quickly while mimicking the pathophysiological development in humans [24]. However, in nude mice, TF2 binding is limited to the grafted human tumor cells, because normal mouse organs do not express human CEACAM5. In humans, TF2 also binds specifically to CEACAM5-expressing tumors, with little binding to normal organs [25]. In a phase I clinical trial, a high tumor/organ ratio (higher than 20) after phenotypic imaging (^111^In-IMP288-TF2) has been demonstrated, highlighting that CEA-expression of normal organs is not a limitation for TF2-pretargeting [26].

^68^Ga-pPET performances was reported in other models of colonic cancer xenografts [13], with tumor uptake of 10.7 +/- 3.6% ID/g in the subcutaneous model, and 23.4 +/- 7.2% ID/g in the peritoneal model. However, this preclinical model is closer to clinical conditions, where tumors are deep, surrounded by liver parenchyma, and more difficult to detect by PET. Tumor uptake depended on tumor size. The major part of the liver tumors analyzed were over 50 mg, since they were easier to detect macroscopically in the liver parenchyma at dissection. Previous biodistribution results of ^68^Ga-IMP288 and ^125^I-TF2 in peritoneal metastases showed an inverse relationship between tumor weight and activity concentration [27], explaining the lower tumor uptake obtained in this study (5.40 +/- 2.72% ID/g).

The use of ^68^Ga in PET imaging is promising, since it has good properties for imaging and it is available from long half-life generators. There are already some promising clinical developments of PET imaging with ^68^Ga, particularly in the imaging of neuroendocrine tumors. ^68^Ga labeling with DOTA compounds improved the diagnosis and management of neuroendocrine tumors compared to previous imaging with somatostatin analogue tracers labeled with ^111^In [28, 29]. Its short physical half-life (68 minutes) requires very fast and efficient targeting. The pretargeting system used here proved adequate, however even better contrasts may have been obtained later after activity injection and in this respect the labeling of di-HSG peptides with ^18^F, which is also feasible [13], or the use of positron emitters with slightly longer half life, such as scandium-44 or copper-64, could improve tumor detection.

The high activity measured in tumors with this pretargeting system paves the way for therapeutic applications. Indeed, replacement of ^68^Ga by alpha- or beta-emitters enables radioimmunotherapy. Indeed, a phase I trial of pretargeted radioimmunotherapy with TF2/^177^Lu-IMP288, after confirmation of tumor targeting with TF2/^111^In-IMP288, demonstrated the feasibility and safety of pretargeted radioimmunotherapy in patients with CRC [26]. A therapeutic phase I/II trial with TF2/^90^Y-IMP288 has been also started in France (EudraCT number: n° 2014-001871-29).

Imaging is a mandatory first step of pretargeted radioimmunotherapy, allowing adapting the injected activity to each patient for a theranostic approach in the context of personalized medicine. Our study demonstrated a good correlation between uptakes analyzed by Inveon software on PET images and activities measured with biodistribution. However, for dosimetric purposes, longer half-life PET emitters should be considered, in particular ^64^Cu (t1/2= 12.7 h). Furthermore, imaging could be used a biomarker to guide antibody-based therapies. Until now, only analyses from tumour biopsies or blood samples can be used as predictive markers for response to therapies. MAbs can be labelled with radionuclides, offering a noninvasive solution to quantitatively assess *in vivo* target expression, whole-body mapping of tumour cell biomarker expression, to select patients for expensive and potentially toxic therapies [30].

The Dock-and-Lock technology enables the production of various antibodies specific to other antigenic targets. For example, in pancreatic carcinoma, TF10, a bispecific anti-PAM4 (expressed by pancreatic carcinoma) and anti-HSG was developed for nuclear imaging and radioimmunotherapy, and where radiation dose estimates suggested that TF10/^90^Y-peptide pretargeting would provide a greater anti-tumor effect compared to ^90^Y-IgG [31]. A radioimmunotherapy trial was also performed in prostate cancer with a bispecific anti-TROP-2 (expressed by prostate cancer cells) and anti-HSG, called TF12, and showed a higher median survival following 2 or 3 cycles compared to controls (>150 vs. 76 days) [32].

In conclusion, ^68^Ga-pPET was more accurate than ^18^FDG-PET for the detection of human colonic cancer liver metastases in a murine model. According to its high sensitivity and the good correlation between PET images and tumor deposition, this imaging method should be further explored as both a diagnostic method and possibly also a more specific approach to radioimmunotherapy. A clinical study evaluating ^68^Ga-IMP288 PET after TF2-pretargeting for the assessment of liver metastases before surgical resection in patients with metastatic colorectal cancer is running, and should help determine its potential in a new diagnostic algorithm for cancer immunodetection.

## MATERIALS AND METHODS

### Cell line

LS174T is a human colon adenocarcinoma cell line (ATCC: CL-188, Rockville, MD), obtained from the American Type Culture Collection, which strongly expresses CEA (appendix 1). LS174T are selected stably transfected cells with the luciferase expressing pCMV-Luc+-SVNeo gene, which codes for luciferase (Inserm U540, Molecular and Cellular Endocrinology of Cancers Unit, Montpellier, France), thus allowing tumor growth visualization by *in vivo* bioluminescence.

### Animal model

The study was approved by the Ethics Committee of French Ministry of Higher Education and Research (reference 00143.01). Female nude mice (NMRI-nu (nu/nu); JANVIER, Le Genet St Ile, France; 10 - 12 weeks old, weight 25-35 g) were housed under standard conditions (standard diet and water ad libitum). Mice were anesthetized by intra-peritoneal injection of a ketamine and xylazine hydrochloride mixture [25 mL of 10 mg/mL Ketalar® (Sandoz), 3 mL of 2% Rompun® (Bayer), and 10 mL of PBS], at the dose of 0.1 mL per 10 g of mouse. One million cells suspended in 0.1 mL sterile physiologic serum were injected into the portal vein through a 30.5 G needle after a short median incision [24].

### Bioluminescence imaging

**Figure 7:**
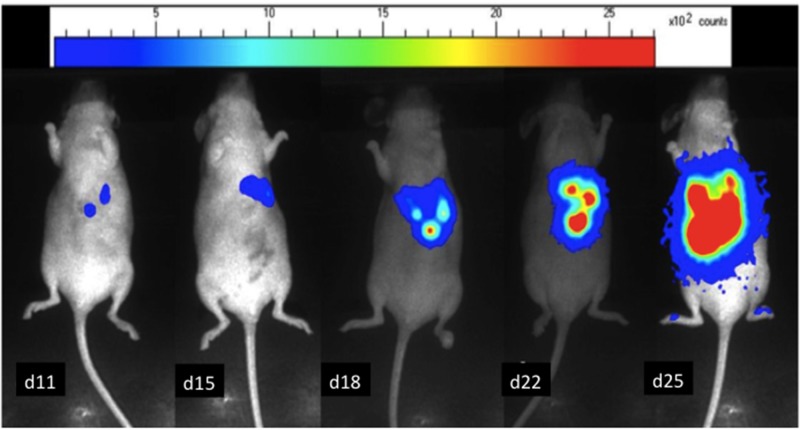
*In-vivo* bioluminescence images of a mouse bearing LS174 Luc+ liver metastases The relative intensity of the photon counts per pixel is represented in color, from the least intense violet blue to the highest red. Tumor progression over time from day 11 to day 25.

After cell grafting, tumor growth was investigated by bioluminescence at day 7 and then every 4 to 5 days. The mice were anesthetized by an intraperitoneal injection of 0.2ml of the anesthetic. Eight minutes after intra peritoneal injection of D-luciferine (1.2 mg, FluoProbes®, Interchim Montluçon, France), photons emitted were collected over 2 minutes, for each animal separately, with an ultra-sensitive CDD camera (PhotonImager®, Biospace) under general anesthesia. A Pseudo-color image was generated, representing light intensity according to a blue to red color-scale. The number of photon counts detected per minute (cpm) for each mouse in a similar region of interest (ROI) was registered to compare tumor growth between animals and over time with the Photovision+ software (Biospace) (Figure 7). PET imaging was performed when the 100,000 cpm level was reached, confirming tumor burden and corresponding to total weight of macroscopic nodules of 150 mg on previous published data [24].

### Animal groups

#### Groups for experimental conditions

To compare ^68^Ga-pPET to ^18^FDG-PET, 3 groups of animals were constituted after tumor grafting and growth control by bioluminescence imaging. The «^68^Ga-pPET» group (n=8) was explored by PET after TF2-pretargeting and injection of ^68^Ga-IMP288 (^68^Ga--PET). The «^18^FDG-PET» group (n=8) was explored by standard ^18^FDG-PET. The «2 imaging» group (n=7) was first explored by ^68^Ga-pPET and 48-72 hours later, by ^18^FDG-PET.

### Negative controls

One healthy animal was explored by ^68^Ga-pPET («control without graft»). One intrahepatic tumor grafted animal was injected with ^68^Ga-IMP288 without TF2-pretargeting («control without TF2»). Data from six mice with subcutaneous tumors of another model of medullary thyroid carcinoma with similar ^68^Ga-pPET and ^18^FDG-PET protocols were also compared for biodistribution data of healthy tissues.

### Pretargeting procedure

TF2 and IMP288 were kindly provided by Immunomedics, Inc., and IBC Pharmaceuticals, Inc. (Morris Plains, NJ, USA). TF2 is a bispecific trivalent antibody, composed of three Fab fragments assembled by the Dock-and-Lock® method, using the natural binding between the regulatory subunits of c-AMP-dependant protein kinase A and the anchoring domains of A kinase anchoring protein [12, 13]. Two Fab fragments are derived from the humanized monoclonal antibody, hMN-14 (labetuzumab, Immunomedics, Inc.), which has binding specificity for human CEACAM5 (CD66e), and one fragment from the humanized monoclonal antibody h679, which specifically binds the histamine-succinyl-glycine (HSG) hapten. TF2 (156 kDa) has two functional CEACAM5 and one HSG binding sites, and is stable in serum, retaining 98% of its binding activity after 7 days [11].

The IMP288 peptide (DOTA-D-Tyr-D-Lys(HSG)-D-Glu-D-Lys(HSG)-NH_2_) contains two HSG moieties and a single DOTA for metal binding. Labeling was performed in a hot cell for synthesis (Medisystem) just before injection. After purification on a Bondelut StrataX column, ^68^Ga was collected and added to 70 μL of IMP288 (100 nmol/mL) with 2.4 mL of acetate buffer, pH 4.5 (76 mL of 0.3 M acetic acid + 24 mL of 0.3 M acetate). The mixture was heated at 95°C for 10 min and then cooled by addition of 2 mL of ultrapure water. To eliminate free ^68^Ga, purification was done by separation on a SEPAK C18 column, previously activated by 5 mL of ultrapure water and 5 mL of ethanol. After washing the cartridge with NaCl, the peptide was eluted with 25% ethanol. Radiochemical purity was controlled by high performance liquid chromatography (HPLC). The expected radiochemical purity exceeded 95%.

### PET imaging

Twenty-four hours after intravenous injection via the tail vein of 6 nmol of TF2 (190 μL, lot 1205118, concentration 5 mg/mL), 4.7 to 10 MBq of ^68^Ga-IMP288 (0.25 nmol of labeled IMP288) were injected in the «^68^Ga-PET» and «2 imaging» groups. The ^68^Ga-IMP288 dose was determined to reach an optimal TF2/peptide ratio [33]. The mice were then maintained under general anesthesia with 2% isofurane (Forane®, USP, Baxter) in an induction box at 30°C under a heating lamp.

PET images were acquired with an Inveon small animal PET/CT scanner (Siemens Preclinical Solutions, Knoxville, TN). Two mice lying in the supine position were evaluated simultaneously under gas anesthesia. Acquisition started 60 minutes after the radiolabeled peptide injection. CT scanning was performed for anatomical reference (voxel size: 113 μm, 80 kV, 500 μA, exposure time 320 msec). CT images were made with a common cone-beam reconstruction method (Siemens). CT acquisition was followed by PET acquisition (time of acquisition: 20 minutes, spatial resolution 1.6 mm, sections of 0.8 mm). Images were reconstructed using the Inveon Acquisition Workplace software (version 1.2, Siemens Preclinical Solutions, Knoxville, TN) with 3D Ordered Subset Expectation Maximization algorithm followed by a fast Maximum A Priori probability algorithm (OSEM3D/MAP). Images were corrected for attenuation and scattering using the Inveon Acquisition Workplace methods Supplementary Figure 1.

### Normal organs analyses

For each organ, two 2D PET images showing the most intense uptake were selected. A ROI was manually drafted around the organ (for well- limited organs such as heart and kidney) or on the organ (for poorly-limited organs, such as liver, bowel or muscle) in each of the 2 selected images. The mean value (in Bq/mL and %ID/mL) was collected as the «Normal organ» uptake.

### Tumor analyses

For each animal, the highest uptake in the tumor was determined with Inveon Research Workplace software (Inveon, Siemens). A constant ROI of 2 mm^3^ was drawn around the hottest spot. The mean value (in Bq/mL and %ID/mL) was collected as the «Tumor» uptake Supplementary Figure 2.

Mice in «^18^FDG-PET» and «2 imaging» groups were fasted for 12 hours before imaging with free access to water. Five to 12 MBq of ^18^FDG were intravenously injected. PET/CT acquisitions started 60 minutes after ^18^FDG injection. Acquisition, reconstruction protocols, «Normal organ» and «Tumor» uptake analyses were similar to those used for the «^68^GaPET» group.

### PET performance analysis

For each mouse, the tumor/normal organ ratio was calculated to explore contrast resolution and evaluate the detectability of the tumor. Statistical comparison of the ratios was performed using a non-parametric, two-tailed, Mann Whitney test using GraphPad InStat software (version 5.00, GraphPad Software).

PET imaging was compared to bioluminescence that had confirmed hepatic colonization. PET/CT images were blinded and analyzed slide by slide to determine the presence of intra-hepatic lesions. The «number of mice with definite lesions in PET/total number of mice with proven tumors by bioluminescence» ratios were calculated to assess the sensitivity of each PET method. Statistical analyses were performed by the two-sided Chi-square test using GraphPad InStat. For each imaging mode, the detection threshold was the tumor volume over which 100% of tumors were detected.

### Biodistribution analyses

The gamma counter was calibrated according to the manufacturer's instructions for ^68^Ga and ^18^FDG. After a retro-orbital blood sample collection, mice were euthanized by cervical dislocation and dissected after ^68^Ga-pPET for the «^68^Ga-pPET» group and after ^18^FDG-PET for the «^18^FDG» and «2 imaging» groups. All macroscopic tumors and normal organs (sample of macroscopically healthy liver, kidneys, muscle, spleen, skin, bone, heart, lungs, bowel, stomach and tail) were collected, weighed and counted in a gamma counter (counting time: 30 seconds per organ), simultaneously with standards prepared from the injected products. Activity measured in the tail was considered as not distributed to mice and subtracted from the injected activity for analyses. For each organ, the amount of present activity (%ID) was calculated:

%ID= (measured activity in organ (cpm)/weight of organ (g))/(standard activity (cpm)-measured activity in tail (cpm)).

Tumor/normal organ ratios were calculated for each animal. Non-parametric, two-tailed, Mann Whitney test using GraphPad InStat software was used for statistical comparisons of means and ratios.

### Correlation between tumor uptakes in biodistribution and PET imaging

To validate the imaging process, a correlation test was performed to assess the relationship between tumor uptake measured in biodistribution analyses (cpm per gram of tumor) and the uptake obtained with PET by Inveon software (in Bq/mL). The selected uptake in PET imaging was the mean value of overall tumor volume, decreased by the natural decay. The non-parametric Spearman's correlation test using GraphPad InStat software was used for the statistical analyses. For all statistical analyses, *P* ≤ 0.05 was considered to indicate a significant difference.

## SUPPLEMENTARY MATERIALS FIGURES


