# Supplementary material for: Sensitivity of pretargeted immunoPET using 68Ga-peptide to detect colonic carcinoma liver metastases in a murine xenograft model: Comparison with 18FDG PET-CT
Source: Oncotarget. 2018 Jun 8;9(44):27502–13. doi: 10.18632/oncotarget.25514 (PMC6007947; doi:10.18632/oncotarget.25514)
Supplement: Supplementary file 1 [file oncotarget-09-27502-s001.pdf]

Sensitivity of pretargeted immunoPET using ^{68}Ga-peptide to detect colonic carcinoma liver metastases in a murine xenograft model: Comparison with ^{18}FDG PET-CT

SUPPLEMENTARY MATERIALS

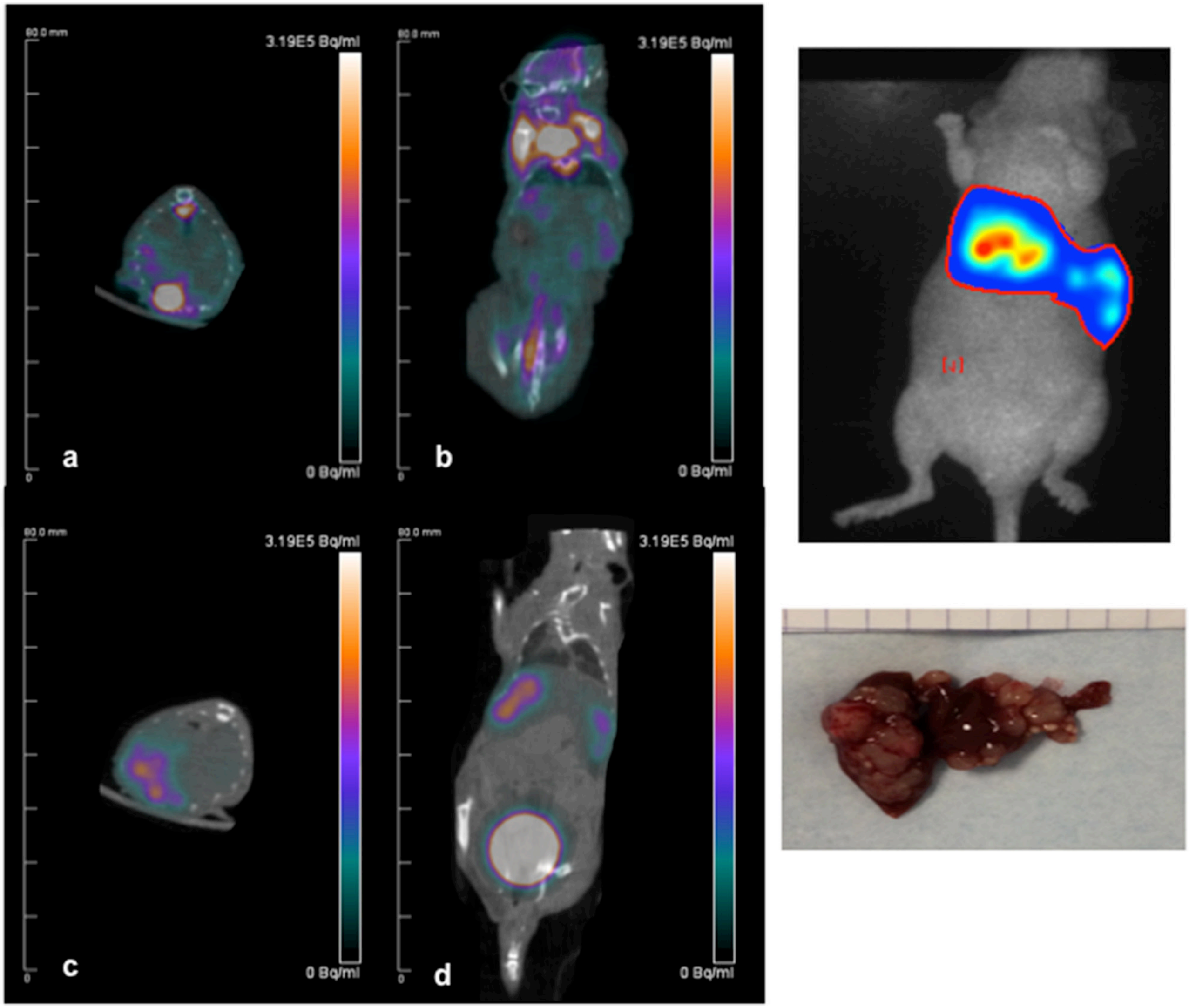

Supplementary Figure 1: PET imaging of hepatic LS174T metastases. ^{18}FDG PET-CT **(a, b)**. Images obtained 1h after intravenous injection of 13 MBq of ^{18}FDG in axial (a) and coronal (b) view. Corresponding ^{68}Ga-pPET-CT **(c, d)**. Images obtained 1 hour after IV injection of 10.1 MBq of pretargeted ^{68}Ga-IMP288. Corresponding bioluminescence imaging and photography after dissection.

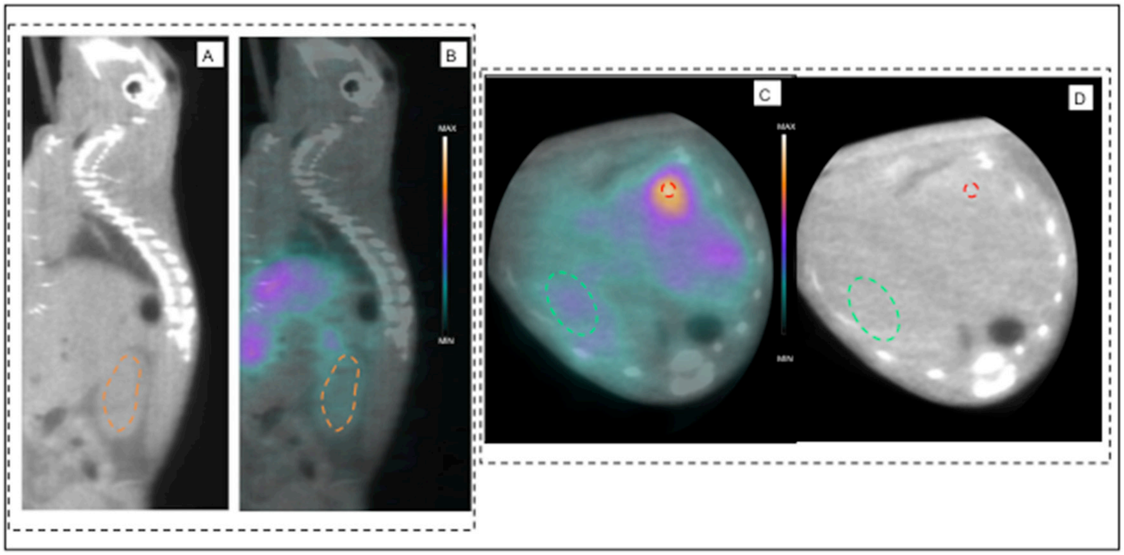

Supplementary Figure 2: ROI measurements on 2D PET imaging. For normal organs, values are obtained from a ROI drafted around the organ (kidney in orange **(A, B)** or on the organ (for poorly-limited organs, such as liver in green **(C, D)** in each of the 2 selected images (most intense uptake). For tumors (c,d), the highest uptake in the tumor is obtained from a constant ROI of 2 mm^3 drawn around the hottest spot (in red).
